# Ultrasmall epibiont *Nanosynbacter lyticus* strain TM7x and host bacteria transcriptional activity after initial host parasitism

**DOI:** 10.1080/20002297.2023.2287349

**Published:** 2023-11-30

**Authors:** Erik L Hendrickson, Batbileg Bor, Kristopher A. Kerns, Lujia Cen, Wenyuan Shi, Xuesong He, Jeffrey S McLean

**Affiliations:** a Department of Periodontics, University of Washington, Seattle, WA, USA; b Department of Microbiology, The Forsyth Institute, Cambridge, MA, USA; c Department of Oral Medicine, Infection and Immunity, Harvard School of Dental Medicine, Boston, MA, USA; d Department of Oral Health Sciences, University of Washington, Seattle, WA, USA; e Department of Microbiology, University of Washington, Seattle, WA, USA

**Keywords:** Transcriptome, RNAseq, oral, Saccharibacteria, epibiont, parasitism

## Abstract

**Background:**

Oral Saccharibacteria *Nanosynbacter lyticus* strain TM7× lives as an ultrasmall epibiont on the surface of its host, *Schaalia odontolytica* strain XH001. Establishing this interaction is a poorly understood multi-step process. The recovery phase marks a shift in the TM7×/host interaction, switching from the early killing phase, with extensive host cell death, to a stable symbiosis phase where the host and epibiont can grow together.

**Results:**

Transcriptomes of TM7× and host, XH001, were captured during the recovery phase and compared to uninfected host and the early host/epibiont interaction (initial encounter). XH001 showed increased expression for rhamnose cell wall components and for the precursor to peptidoglycan while TM7× showed increases in the peptidoglycan pathway. Transporter expression was generally increased for both organisms during recovery compared to the initial encounter, though, XH001 showed lower amino acid transporter expression. Consistent with host parasitism, XH001 showed increased expression of various stress-related genes during recovery while TM7× showed reduced stress. TM7× displayed higher expression of type IV pili, consistent with increased attachment to new hosts.

**Conclusion:**

As TM7× is a member of the broadly distributed Candidate Phyla Radiation with small genomes lacking numerous biosynthetic pathways, this study provides further insights into how these epibionts interact and modulate their host bacteria.

## Introduction

The Saccharibacteria *Nanosynbacter lyticus* (*Nl*) strain TM7× is unique in the human microbiome. Isolated from the oral cavity, it has an ultrasmall cell size (200-300 nm) and a small genome lacking numerous biosynthetic pathways. It forms a dynamic relationship with a bacterial host named *Schaalia odontolytica* strain XH001, formerly *Actinomyces odontolyticus* strain XH001, as an epibiont [1,2]. This epibiont lifestyle, living on the surface, significantly modulates host activity. Previous findings suggest that TM7× has a multi-step mechanism for establishing symbiosis with its host bacteria. When TM7× is introduced to XH001 that has not previously been exposed to TM7×, known as naïve XH001 or XH001n, there is an initial encounter between epibiont and host, which we term the initial encounter phase. This is followed by a killing phase accompanied by a significant drop in host numbers and a subsequent recovery phase leading to the establishment of a stable association between epibiont and host, referred to as stable symbiosis [3]. The ability of free-floating TM7× to infect a new host horizontally [3] involving type IV pili [4] also suggests that this mechanism may have a role in the dissemination and persistence of Saccharibacteria in the human oral cavity [3,4].

This epibiont lifestyle is likely shared across numerous ultrasmall bacteria. *Nl* TM7× is the first cultivated species of the Candidate Phyla Radiation (CPR) group, a monophyletic group with small genomes lacking numerous biosynthetic pathways, also referred to as the Patescibacteria super-phylum [1,5–7]). Despite their reduced genomes, it has recently been discovered that members across the Saccharibacteria display wide genetic diversity and are highly prevalent in the human microbiome and other mammals [2,8] [7]. This has piqued interest in understanding the mechanisms underlying their obligate symbiosis.

TM7× falls within the G1 group of Saccharibacteria, recently proposed to encompass the Nanosynbacteraceae and Saccharimonadacea families [7]. This group includes both environmental and mammalian-associated members while remarkably sharing around 60% of the protein-coding genes in their small, biosynthetically restricted genomes and maintaining high gene synteny [7]. TM7× has the most streamlined genome among the fully closed genomes of sequenced isolates from the G1 group [9,10]. The TM7× genome contains 739 predicted genes, of which 692 are protein-coding, with short intergenic regions resulting in 94% coding base counts [1]. It lacks major pathways such as the tricarboxylic acid cycle and de novo synthesis of nucleotides and amino acids, as do other members of the CPR [5,6].

We have previously investigated how TM7× and its host XH001 interact during the establishment and maintenance of symbiosis via transcriptomic profiling, examining the initial encounter and stable symbiosis [11]. There were dynamic shifts in gene expression for both species, with significant concordance to proteome expression across many genes, suggesting that the interaction is complex and requires constant adjustments to maintain a balance. The increased expression of peptidoglycan biosynthesis, mannosylation, cell cycle, and stress-related genes in XH001 during stable symbiosis imply that the host is investing more energy in cell wall production and stress response, perhaps to counteract the presence of the epibiont on its surface. During the interaction, XH001 cells infected with TM7× develop an elongated cell shape and thickened cell walls, consistent with this hypothesis [11,12]. On the other hand, TM7× showed increased expression of pili, type IV effector genes, and arginine catabolism/biosynthesis genes during stable symbiosis, suggesting that these functions play a crucial role in the interaction. The higher levels of energy production and peptidoglycan biosynthesis in TM7× during stable symbiosis, both expected to require host-derived metabolites, are consistent with its obligate epibiotic lifestyle.

The recovery phase marks an important shift in the TM7×/host interaction, switching from widespread killing of the host XH001 cells to an interaction where the host can survive and grow in the presence of epibiont, necessary for stable symbiosis. It is unknown whether this shift occurs as a result of phenotypic adaptation in the host, TM7×, or both. *S. odontolyticus* could develop resistance or tolerance to TM7× or adopt phenotypic alterations enabling growth. In contrast, TM7× cells may adapt to allow growth without overwhelming the host. In order to help understand this dynamic relationship of relevance to what is occurring in the human oral cavity, we have performed RNAseq on this recovery phase with high temporal sampling.

## Materials and methods

### Bacterial strains and growth conditions

Naive XH001 (XH001n) (*Schaalia odontolytica*, formerly *Actinomyces odontolyticus* subsp. *actinosynbacter* strain XH001) monoculture that had not previously been exposed to TM7× and XH001/TM7× coculture were both grown under optimal conditions for XH001 [12], Bacto Brain Heart Infusion (BHI, BD) broth at 37°C in a microaerophilic chamber (2% O2, 5% CO2, balanced with N2) [3,13]. To avoid sample contamination, the cultures were inoculated and passaged outside the microaerophilic chamber under sterile aerobic conditions. Brief aerobic exposure does not affect XH001 growth. Free-floating TM7× cells (*Nanosynbacter lyticus* Strain TM7× HMT-952) were isolated from XH001/TM7× coculture and quantified using a modified virus counting assay as described previously [3]. Briefly, XH001/TM7× coculture cells were filtered through 0.45 μm filters. TM7× cells in the filtrate were collected by ultracentrifugation at 80,000 × g for 90 min. Only freshly isolated TM7× cells, without a freeze-thaw cycle, were used for infection experiments.

### TM7× infection, passaging, and sample collection

**Figure 1.**
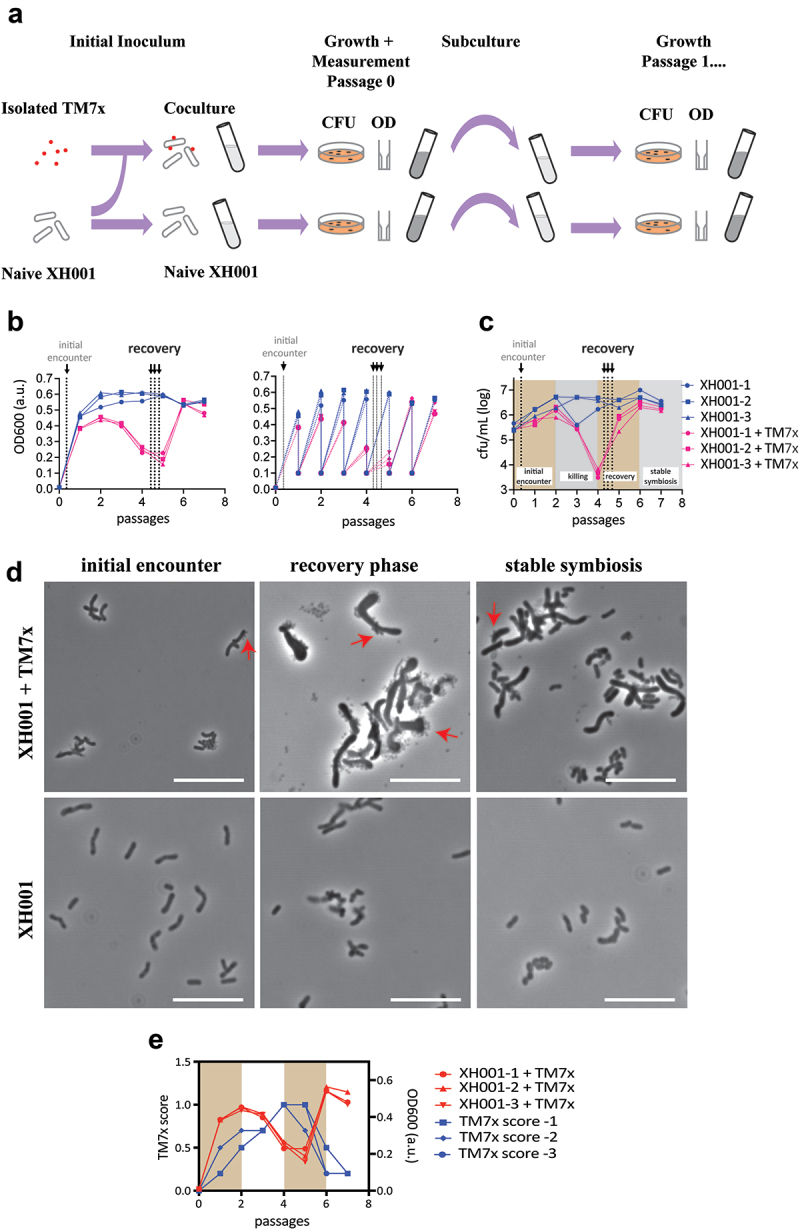
Experimental design. a) reproduced from [11], a schematic of culture setup, growth, and measurement for the naïve and cocultures. Red dots are TM7× while XH001 are shown as rod shaped tubes. Optical density at 600 nm (OD600) and colony forming units (CFU) were quantified at each step of the passage. OD600 was used to determine dilution to start each passage and represented in panel b. CFU is shown in panel c. b) growth measurements for passages using OD600 over seven passages (x-axis) showing the OD600 for the end of each passage (left). The right graph is identical, but additionally shows the diluted OD600 measurements at the start of each passage. Three individual XH001n cultures are shown in blue circles, squares and triangles, while cultures of XH001n with TM7× added are shown in red circles, squares and triangles. Sampling points for the initial encounter phase (passage 0 at 6 hours) and recovery phase (passage 4 at 6, 10, and 15 hours) for the transcriptomics sequencing are indicated by arrows and dashed lines. c) growth measurements as determined by CFU over seven passages (x-axis). Three individual XH001n cultures are shown in blue circles, squares and triangles, while cultures of XH001n with TM7× added are shown in red circles, squares and triangles. Sampling points for the initial encounter phase (passage 0 at 6 hours) and recovery phase (passage 4 at 6, 10, and 15 hours) for the transcriptomics sequencing are indicated by arrows and dashed lines. The CFU measurements were used to determine the phases of the interaction and are indicated by colored backgrounds. d) phase-contrast images of XH001n and XH001n/TM7× during the initial encounter (passage 0 at 6 hours), the recovery phase (passage 4 at 6 hours), and stable symbiosis (passage 6 at 6 hours) are shown. All scale bars are 10 µm. e) XH001n/TM7× OD600 in panel b plotted alongside TM7× score, giving a qualitative measurement of TM7× presence on the host cells. High scores indicate that the majority of host cells showed extensive number of TM7× on their surface. Low scores represent no or minimal number of TM7× on the surface [3]. Initial encounter and the recovery phases are indicated by colored backgrounds.

Infection and passaging were conducted as previously reported [11]. In brief, XH001n cultures were started from frozen stock and passaged twice in BHI media every 24 hours to ensure homogeneous cultures in similar bulk growth states. 115 mL of stationary phase XH001n cells were mixed with free-floating TM7× cells in a 1:1 cell ratio in triplicate and grown in BHI medium. At the end of each passage, the 24-hour mark, XH001 cell density was measured with both OD600 and colony forming units (CFU). Cultures were then passaged into fresh media diluting to 0.1 OD600 (Figure 1a). For the recovery phase, samples were taken during the fourth passage at three different time points, 6, 10 and 15 hours into passage 4 (Figure 1b). Triplicate controls were run by taking XH001n, without the addition of TM7×, through the same inoculation, passaging, and sampling. For each sample, 50 mL of culture were removed and centrifuged at 13,000 × g for 10 minutes at 4°C, discarding the supernatants. The pellets were flash-frozen in liquid nitrogen. Phase-contrast microscopy was used throughout to ensure the purity of our cultures. Because OD600 and CFU capture only XH001, microscopy was used to obtain a qualitative measurement of TM7× presence on host cells via TM7× score [3] (Figure 1d). A TM7× score was determined by phase-contrast microscopy with scores ranging from 0.2 for one or two host cells with TM7× to 1 for the majority of host cells decorated with large numbers of TM7× as well as free-floating TM7×.

### RNA isolation and sequencing

RNA was isolated as previously described [11]. Briefly, cell pellets were resuspended in cold PBS buffer and lysed by bead beating, 3 times for 30 seconds at 6 m/s with 30 second breaks between. Total RNA was isolated using the High Pure RNA isolation kit (Ref#11828665001), cleaned and concentrated with Zymo Research RNA clean & concentrator kit following kit protocol (Cat#R1015), and the DNA removed using the Ambion TURBO DNA-free kit (Cat#AM1907). RNA concentrations were measured with an RNA broad range Qubit kit and Nanodrop and Qubit 3 used to determine nucleic acid purity. Ribozero rRNA Removal Kit (Illumina, South Plainfield, NJ, USA) was used to deplete rRNA. The libraries were sequenced using an Illumina HiSeq platform by GENEWIZ, LLC. (South Plainfield, NJ, USA). The paired-end reads were trimmed of low-quality sequences at a quality cutoff of 30 using BBDuk 38.37 [14] and mapped to a reference database containing XH001 and TM7× reference sequences using Geneious prime (https://www.geneious.com) with the default settings. An average of 24 M reads were mapped to XH001 and an average of 11.5 M to TM7×.

### Bioinformatics

Degust [15] was used to calculate expression ratios and false discovery rates (FDR) using voom/limma [16] with a significance cutoff of FDR 0.05 and a ratio of 0.5 or more in either direction using the triplicate biological replicates. Annotations were assigned as previously described [11]. Categories of orthologous genes (COG’s) were obtained from eggNOG [17] and pathways from the KEGG database [18,19]. Results for all genes for XH001 and for TM7× are given in **Supplemental Table 1** (see Data Availability).

## Results and discussion

### Experimental design and RNA sequencing

Samples for RNA sequencing were produced by infecting a naive XH001 strain (XH001n), a strain that was not originally exposed to TM7×, with TM7× at a ratio of roughly 1:1 and passaging longitudinally six times. During each passage the coculture was grown for 24 hours and then subcultured to a starting OD600 of 0.1 (Figure 1a). XH001n controls, without TM7×, were grown through the same passaging regime using the same protocol and time scale. OD600 and colony-forming units (CFU) were used to determine the XH001 numbers at each passage when subculturing (Figure 1b). This only determined host numbers. Neither technique effectively measures TM7× cells, CFU because TM7× cannot grow without a host and OD600 due to the epibiont’s small size [3]. To assess TM7× levels on its host, a qualitative TM7× score was determined by microscopy (Figure 1c,d) [3].

As shown in Figure 1, XH001n exposed to TM7×, though not the unexposed control, underwent a phase where host killing is observed with rapid CFU reduction (passage 2–4) followed by a characteristic host recovery phase (passage 4–6). By passage 6, the XH001n infected TM7× culture attains a stable symbiosis. The observed killing phase (also referred to as crash phase) [3] as reflected by CFU reduction is slightly different from OD600 measurement. As seen in previous studies [3], OD600 drops later than CFU, presumably because CFU only measures viable cells while OD600 also encompasses dead bacteria. As far as our total transcript analysis, we solely focus on the live bacteria determining each phase using the CFU. Passages 4–6 were labeled as recovery phase. TM7× score (Figure 1c, d) agreed with previous studies [3,13] showing that TM7× gradually increases after infection, peaking during the host killing phase, and then slowly decreases through host cell recovery to stable symbiosis.

Samples for both XH001/TM7× coculture and XH001n monoculture were collected from three time points during the recovery phase, passage 4 (6, 10, and 15 hours). Total RNA was extracted, sequenced, trimmed for quality, and mapped to genomes as previously described [11]. XH001 had an average of 24 M mapped reads per sample. TM7× had 11,5 M average reads.

### Functional level changes during the host recovery phase

**Figure 2.**
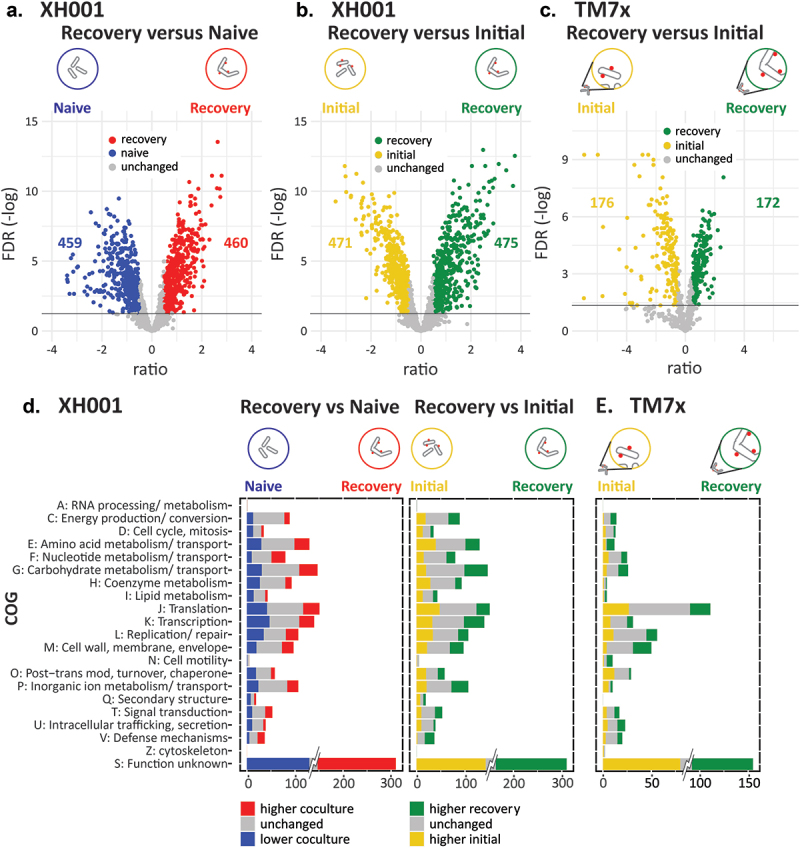
Significant differences between conditions. a) volcano plot of the log2 ratio of expression levels and -log of the FDR. Shown is XH001 recovery phase coculture versus naive. Colored dots indicate genes that made the 0.05 FDR and 0.5 log_2_ ratio cutoffs. Blue: lower in recovery; red: higher in recovery; yellow: higher in initial encounter coculture; Green: higher in recovery phase coculture. The number of significantly differentially expressed genes are shown on the plots. b) XH001 recovery phase coculture versus initial encounter coculture. c) the TM7× comparison of recovery phase coculture versus initial encounter coculture is also shown. d-e) significantly differentially expressed genes for the clusters of orthologous groups are shown for d) XH001 compared to naive and compared to the initial encounter and e) TM7×. The number of unchanged (grey) and significantly differentially expressed genes (colored) for each COG are shown. To prevent the large number of genes in cluster S: unknown function from dominating the scale, only the significant differences are shown at full value. XH001 contains 621 genes annotated as S: unknown function. TM7× contains 260.

Comparisons employed a significance cutoff of FDR 0.05 and excluded results with log_2_ ratios between −0.5 and 0.5. The 10- and 15-hour recovery phase samples showed little difference when compared to the 6-hour sample, indicating that the recovery phase encompasses passage 4 and all time points in passage 4 have a consistent expression pattern. Therefore, the primary analysis was carried out on the 6-hour sample with the others shown in Supplemental Table 1. The 6-hour (Recovery) results are shown in Figure 2.

Changes to the host transcriptome during the recovery phase were examined using two different comparisons. The first compared the XH001/TM7× coculture to the corresponding naive XH001n controls. As seen in Figure 2a, recovery-phase showed 460 (24%) genes with increased expression in the coculture and 459 (24%) genes with decreased expression, indicating a significant change in host expression from the presence of the epibiont. To understand the changes occurring across the interaction, the recovery phase samples were also compared to the early interaction between XH001n and TM7× (Figure 2b), termed the ‘initial encounter’, which has been previously described [11]. For these samples, XH001n exposed to TM7× were collected 6 hours into passage 0 (Figure 1). The result showed 475 (25%) genes with higher expression in the recovery phase and 471 (24%) genes with higher expression during the initial encounter, indicating a substantial shift in XH001 gene expression between these phases. For the TM7× transcriptome (Figure 2c), comparing the recovery phase to the initial encounter also showed a substantial shift in expression with 172 (25%) genes with higher expression in the recovery phase and 176 (25%) genes higher during the initial encounter. These samples were collected and processed from the same experiment as the initial encounter and stable symbiosis samples discussed in our previous paper [11]. As an orthologous check on the differential RNA expression, we compared the transcriptome results to proteomics comparison of XH001 with and without TM7× and found a general correlation between the proteomic and transcriptomic results, providing support for the transcriptome results [11].

In order to characterize the global shift in functional pathways, the results were broken down by categories of orthologous genes (Figure 2d-e). Most categories showed genes with significantly increased and decreased expression, however, some skewed more heavily to one condition. Specifically, compared to the naive control, XH001 cells during the recovery phase showed proportionally more increased gene expression for nucleotide metabolism and defense mechanisms compared to genes with reduced expression (29/10 and 15/5 respectively). A skew towards reduced expression in the recovery phase was seen for cell cycle (13/5), lipid metabolism 14/5(, coenzyme metabolism (27/13), modification (19/8), and intracellular trafficking (11/5). The host cell colony forming units are increasing during the recovery phase [3]. However, previous physiology and single cell imaging studies suggested that super-infected host bacteria, up to 50 TM7× per host bacteria (Figure 1c), within the recovery culture have a lower growth rate compared to naive host [3]. Therefore, a skew towards lower cell cycle genes implies that slow or non-growing host bacteria are reducing overall cell division during the recovery phase.

Comparing XH001 gene expression in the recovery phase to the initial encounter phase showed a predominance during recovery of carbohydrate metabolism (48/20), inorganic ion metabolism (35/21), and defense mechanism genes (20/2). A predominance of higher expression in the initial encounter phase was found for cell cycle (13/6), coenzyme metabolism (29/13), translation (48/27), and intracellular trafficking (10/4). Once again, both cell cycle and replication genes were higher during the initial encounter despite the recovery phase being noted for increasing host cell numbers. However, at initial encounter, XH001 cells are growing rapidly and have not undergone selection by TM7× while the majority of the recovery XH001 cells are infected with many TM7× bacteria (Figure 1c, d).

For TM7×, the categories that skewed towards higher expression during the recovery phase were energy production (6/1), amino acids (8/3), carbohydrates (10/4), cell wall (19/4), cell motility (6/1), and intracellular trafficking and secretion (8/5). More prominent during the initial phase were post-translational modification (12/2) and inorganic ion metabolism (6/2). Most of the categories associated with the recovery phase involve substrates that TM7×, lacking numerous biosynthetic pathways, is incapable of producing de novo and are presumably provided by the host, implying a more effective symbiosis during recovery than in the first few hours of interaction.

### Cell cycle and cell wall/membrane

The presence of TM7× on the host has been shown to inhibit cell division and cell growth, resulting in cell elongation and club-ended morphologies (Figure 1c) [3]. As a result, changes were expected in genes for DNA replication, cell cycle, and cell wall/membrane biosynthesis. However, COG replication and repair (Figure 2d-e) showed similar numbers of increased and decreased genes for all comparisons. Even examined at the individual gene level the results were inconclusive.

**Figure 3.**
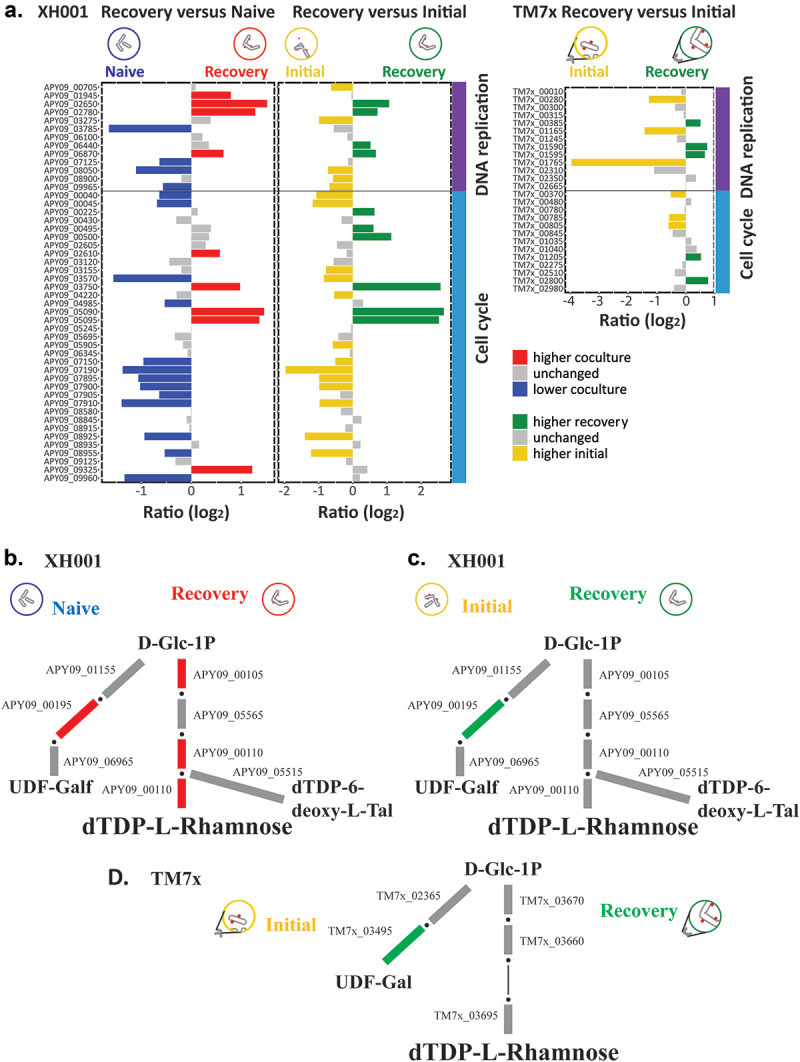
DNA replication, cell cycle, and rhamnose biosynthesis. a) genes for DNA replication and cell cycle. Bar plots show the log_2_ ratio between the recovery phase coculture and naive and between the recovery phase coculture and the initial encounter coculture. Red indicates significantly increased in the recovery phase compared to naive and blue significantly decreased. Green indicates significantly increased during recovery and yellow significantly increased in the initial encounter phase. b-d) a schematic of O antigen sugar biosynthesis pathways for XH001 and TM7×. Due to its small genome TM7× has fewer predicted genes in these pathways. Red: increased in XH001/TM7× versus XH001n; blue: decreased in XH001/TM7× versus XH001n; Green: increased in recovery phase; yellow: increased in initial encounter; Grey: statistically unchanged. b) recovery versus naive. The XH001 APY09 gene designations are given. c) recovery versus initial encounter. d) TM7× recovery versus initial encounter. The TM7× gene designations are given. Steps that currently have no predicted gene but are expected to exist are shown in thin black lines.

In contrast to DNA replication, COG cell cycle (Figure 2d-e) trended towards lower expression during the recovery phase (Figure 3a). Out of 35 genes, XH001/TM7× showed five increased genes compared to naive host. These were associated with chromosome and plasmid partitioning including a chromosome partitioning protein (APY09_02610), prevent host death protein (APY09_03750), plasmid stabilization protein (APY09_05090), and an anti-toxin (APY09_05095). 13 genes showed lower expression compared to the XH001n alone control, including *ftsX*, *ftsE, ftsW*, and *ftsK* (APY09_00040, APY09_00045, APY09_08955, APY09_09960), cell division protein DivVA (APY09_07190), *sepF* (APY09_08925), a death on curing protein (APY09_04985), a chromosome partitioning protein (APY09_07905), and segregation and condensation proteins A and B (APY09_07900, APY09_07895). These results were consistent with the idea that bacterial DNA replication, cell division, and cell growth are related but separate processes [20].

Compared to the initial encounter phase, 6 genes had higher expression while 13 genes were higher in initial encounter phase. Higher in recovery included prevent host death protein (APY09_03750), plasmid stabilization protein (APY09_05090), and an anti-toxin (APY09_05095). Higher during the initial encounter included *ftsX*, *ftsE*, and *ftsW* (APY09_00040, APY09_00045, APY09_08955), cell division protein DivVA (APY09_07190), *sepF* (APY09_08925), anti-toxin PHD (APY09_09505), and segregation and condensation proteins A and B (APY09_07900, APY09_07895). Together, these results indicate lower cell division during the recovery phase. As mentioned, higher TM7× scores during the recovery phase (Figure 1d) indicate more infected cells and could explain the difference.

Between the two phases, TM7× showed fewer differences amongst cell cycle genes. Out of 13 genes, only 2 were higher during recovery though one was *ftsA* (TM7×_02800). Three were higher during the initial encounter including *ftsK* and *ftsZ* (TM7×_00370, TM7×_00785). This is consistent with the idea that while TM7× may enjoy enhanced growth during the killing phase (Figure 1d), as the host recovers, TM7× may need to adapt to the recovered host by reducing its growth.

Looking at peptidoglycan biosynthesis showed no consistent changes in the XH001 pathway for either comparison. However, APY09_05200 UDP-N-acetylglucosamine diphosphorylase showed higher levels in recovery than XH001n and the initial encounter. This enzyme produces the peptidoglycan precursor UDP-N-acetylglucosamine (UDP-GlcN Ac). TM7× cannot synthesize UDP-GlcN Ac, but all of the TM7× genes leading from UDP-GlcN Ac to peptidoglycan showed higher levels during recovery. This was consistent with higher UDP-GlcN Ac production in XH001 and potential transfer to TM7× for its peptidoglycan production.

COG cell wall, membrane, and envelope biogenesis (Figure 2d-e) did not show a significant skew. However, rhamnose is present in the cell walls of both XH001 and TM7× [1,12]. As seen in Figure 3b-d, the pathway to dTDP-L-rhamnose was higher compared to XH001n implying increased rhamnose in the cell wall during the recovery phase. Both XH001 and TM7× also showed higher levels in recovery for UDP-glucose 4-epimerase *galE* (APY09_00195, TM7×_03495) in all comparisons for a potential increase in UDP-galactose.

### Energy metabolism

**Figure 4.**
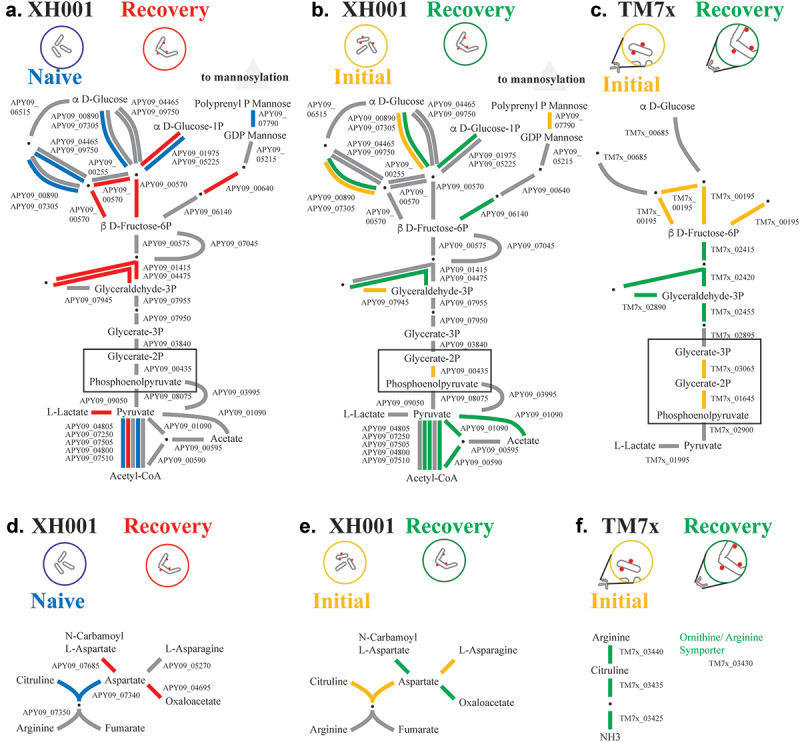
Glycolysis and arginine. a-c). A schematic of the glycolysis pathway and polyprenol phosphate mannose for XH001 and TM7×. Due to its small genome TM7× has fewer predicted genes in these pathways. Steps with alternative pathways are shown as separate connections. Steps with multiple subunits are shown with multiple lines. Red: increased in XH001/TM7× versus XH001n; blue: decreased; Green: increased in recovery; yellow: increased in initial encounter; Grey: statistically unchanged. Boxes are drawn to highlight certain genes. A) recovery versus naive. The XH001 APY09 gene designations are given. b) recovery versus initial encounter. c) TM7× recovery versus initial encounter. The TM7× gene designations are given. D-F) a schematic of the arginine pathway for XH001 and TM7×. d) recovery versus naive. The XH001 APY09 gene designations are given. e) stable symbiosis versus initial encounter. f) TM7× stable symbiosis versus initial encounter. The TM7× gene designations are given.

COG energy production and conversion (Figure 2d-e) showed similar numbers of genes with increased and decreased expression in both XH001/TM7× comparisons though, as expected, a skew towards higher levels during recovery for TM7×. A more detailed look at glycolysis is presented in Figure 4a-c. XH001 in the recovery phase compared to XH001n and the initial encounter phase showed a mixture of increased and decreased expression between glucose and glyceraldehyde 3P. However, compared to naive, recovery seemed to shift pyruvate conversion from acetyl-CoA to L-lactate while compared to the initial phase recovery favored acetate and acetyl-CoA (Figure 4a-b). Enolase (APY09_00435) was lower in recovery compared to initial phase. Interestingly, TM7× showed higher expression between D-fructose 6P and glycerate 1,3P, but lower expression of 2,3-bisphosphoglycerate-dependent phosphoglycerate mutase (TM7×_03065) and enolase (TM7×_01645) during the recovery phase (Figure 4c). Previous analysis of stable symbiosis showed overall increased glycolysis genes for TM7× compared to the initial encounter except for reduced levels of that same mutase (TM7×_03065) [11]. Interestingly, 2,3-bisphosphoglycerate-dependent phosphoglycerate mutase (APY09_03840) was the one gene with increased expression in the stable symbiosis host compared to XH001n. The unusual patterns of expression regarding these two central elements of energy metabolism do not offer a straightforward explanation. However, changes in multiple experiments imply an important role for these genes in the epibiont interaction.

### Arginine

A possible alternative source of energy for TM7× is arginine [21]. Despite CPR species being noted for their missing de novo amino acid biosynthesis pathways [5,6], within the Saccharibacteria, the mammalian-associated members have acquired the four gene locus for complete arginine catabolism [21]. TM7× has been shown to utilize arginine as an energy source even in the absence of its host. XH001 does not derive energy from arginine but can produce citrulline which can be utilized by TM7×. In TM7× both the arginine pathway and the ornithine/arginine symporter (TM7×_03430) showed higher expression during recovery (Figure 4f). However, while XH001 showed higher expression through aspartate during recovery, the pathway to arginine production from citrulline appeared lower during recovery (Figure 4d-e), indicating that TM7× may not be inducing this particular pathway for increased arginine from XH001.

### Secretion systems and appendage genes

**Figure 5.**
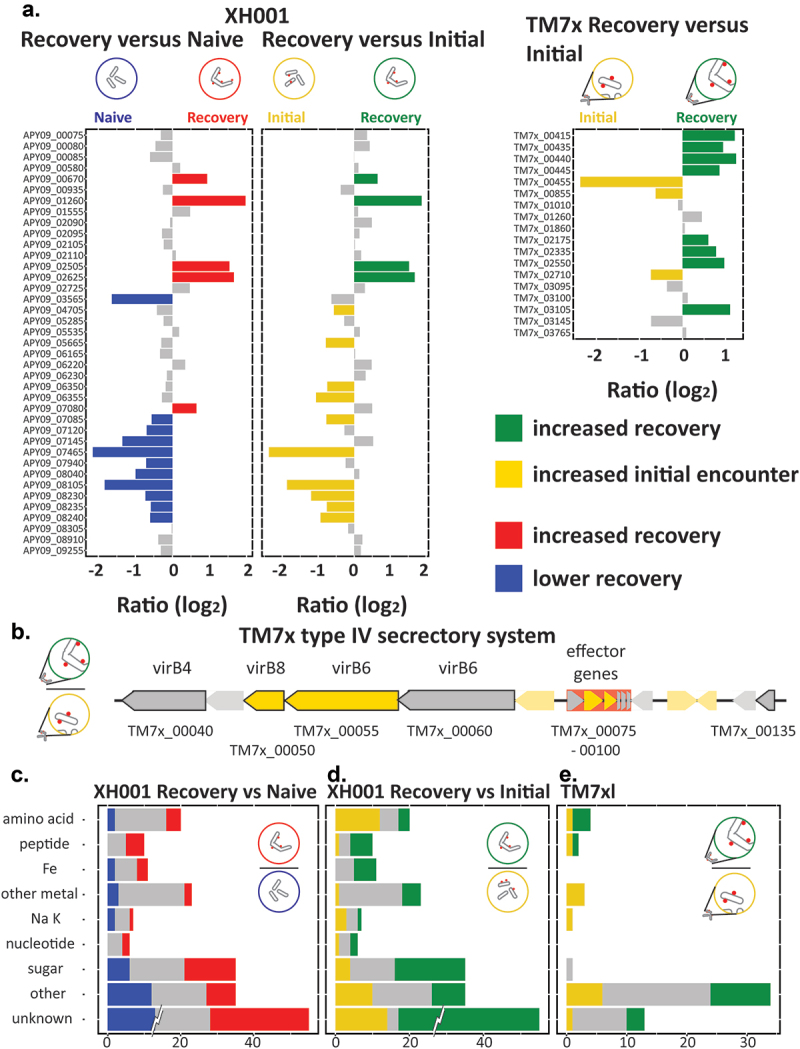
Secretory systems and transporters. a) genes for secretory systems, excluding the type IV system from TM7×. Bar plots show the log_2_ ratio between the stable symbiosis coculture and the initial encounter coculture. Blue: decreased in XH001/TM7× versus XH001n; Green: increased in recovery; yellow: increased in initial encounter; Grey: statistically unchanged. b) a schematic of the type IV secretory system region of TM7×. Black outlines indicate predicted type IV component genes. Putative effector genes are backed in red. Green indicates significantly increased during recovery and yellow increased in the initial encounter. c-e) transporters. The number of unchanged and significantly differentially expressed genes for transporter groups are shown. Groups: amino acids, peptides, iron (fe), non-iron metal transporters, sodium/potassium (na/K), sugar, other, and genes predicted to be transporters but without an identifiable substrate (unknown). To prevent the large number of XH001 genes in unknown, 95, from dominating the scale, only the significant differences are shown at full value. c) recovery versus naive control. d) recovery versus initial encounter. e) TM7× recovery versus initial encounter.

Nutrient transfer is likely to play a significant role in the epibiont interaction. Interestingly, COG intracellular trafficking and secretion trended downward during recovery compared to XH001n and the initial encounter (Figure 2d-e). As shown in Figure 5a, compared to XH001n, 5 genes were higher during recovery including signal recognition particle-docking protein *ftsY* (APY09_07080) but 11 were reduced during recovery including signal peptidase (APY09_07120), a signal recognition particle protein (APY09_07085), prepilin peptidase (APY09_08105), and s*ecD*, s*ecF*, and *secG* (APY09_08235, APY09_08230, APY09_07940).

Comparing the recovery phase to the initial encounter yielded the same four genes higher in recovery as seen in the naive comparison with *ftsY* no longer making the significance cutoff. 10 genes had higher expression during the initial encounter including the signal recognition particle protein (APY09_07085), prepilin peptidase (APY09_08105), both *clpP* (APY09_06350, APY09_06355), preprotein translocase subunit *yajC* (APY09_08240), and *secA*, *secD*, s*ecE*, and *secF* (APY09_05665, APY09_08235, APY09_04705, APY09_08230). Taken together these implied a reduction in sec pathway export during recovery.

In contrast to its host, TM7× COG intracellular trafficking and secretion trended upwards during recovery (Figure 2d-e). 8 of the 18 genes showed increased expression during recovery (Figure 5a). These included pilin subunit genes, both *pilM* and *pilC*, and twitching motility protein *pilT* (TM7×_00415, TM7×_03105, TM7×_00445, TM7×_00440), as well as one of the signal peptidase I’s (TM7×_02175), type II secretion system protein E (TM7×_00435), and general secretion pathway protein *gspE* (TM7×_02335). Only the other signal peptidase 1 (TM7×_00855), prepilin signal peptidase like protein (TM7×_00455), and *secG* (TM7×_02710) showed higher levels during the initial encounter. These changes indicated that TM7× appendage related genes are crucial during the recovery phase. In the Saccharibacteria strain TM7i, the pili system has been shown to be responsible for twitching motility and important for locating and associating with host cells [4]. Similar mechanisms and utility may be important for TM7× cells in our experiments. These pili are widely conserved within the Saccharibacteria, and while it has not been confirmed by functional studies, TM7× likely displays the same motility. Increased pili expression would be consistent with spreading to the increasing host numbers during recovery. Figure 5b shows the previously identified unique type IV secretion system with putative predicted effector genes for TM7× [7]. Two of the system components (TM7×_00050, TM7×_00055) and two of the putative effector genes (TM7×_00080, TM7×_00085) showed higher levels during the initial encounter, implying a greater role earlier in epibiont association.

### Transporters

The putative transporter genes in XH001 were divided into groups based on possible substrate specificity [11]. Compared to naive host, most groups showed relatively few significant differences. Large differences were seen in sugar, peptides, and other transporters. For sugar, 14 transporters had higher expression during recovery and 6 had lower expression. Five of the 10 putative peptide transporters were higher during recovery. Other transporters with increased recovery phase expression included phosphate transport proteins (APY09_01710, APY09_01715), macrolide transporters (APY09_01600, APY09_02985), and a glycerol transporter (APY09_01260). The decreased genes were primarily cell division and SEC genes mentioned earlier and two chloride channel proteins (APY09_03815, APY09_08465).

Comparing the recovery phase to the initial encounter showed elevated transport during recovery, with 38% of the predicted genes having higher expression during recovery (Figure 5c), but only 19% higher during the initial encounter. Peptide transporters, sugar transporters, and iron and other metal transporters were generally increased during recovery. Interestingly, one group, amino acid transporters, showed higher levels during the initial encounter. Other genes increased during recovery included autoinducer Ai-2 binding protein (APY09_02520), a macrolide transporter (APY09_01600), a glycerol transporter (APY09_01260), and two multidrug ABC substrate binding proteins (APY09_09145, APY09_09150). Other transporters that showed higher expression during the initial encounter were primarily cell cycle and secretion genes previously mentioned as well as a couple of chloride channel proteins (APY09_03815, APY09_08465). Overall, transport appeared to be increased during recovery, consistent with increased recovery and uptake of nutrients. The exception was the group of amino acid transporters, which had higher expression during the initial encounter.

TM7× also showed a skew towards higher transporter expression during recovery with 17 genes higher in recovery and 13 higher during the initial encounter (Figure 5c). Those with higher recovery phase expression genes were 3 of the 4 amino acid transporters including the arginine/ornithine symporter (TM7×_03430, TM7×_03480, TM7×_03485), a peptide transporter (TM7×_03475), H(+)-transporting ATPase (TM7×_01440) and lipid A export permease (TM7×_02745). Higher during the initial encounter were the secretion genes mentioned previously, some peptide transporters (TM7×_00200, TM7×_02705), all the predicted metal transporters (TM7×_01845, TM7×_03415, TM7×_03420), a sodium transporter (TM7×_01545), and a macrolide transporter (TM7×_00205). The role of traditional transporters in nutrient transfer from the host is unclear. The reduction in metal transporters could be a shift from scavenging the environment to host transfer. In contrast, amino acid transporters went up during recovery for TM7×, but down compared to the initial encounter for XH001. The increased expression would be consistent with increased access to amino acids expected from host association. However, the data did not indicate higher arginine production for XH001 despite higher recovery phase expression of the arginine/ornithine symporter in TM7×. The amino acid transporters could be for environmental sources, as is seen with TM7× utilizing arginine as an energy source in the absence of its host [21], with lower host levels reducing competition for solubilized nutrients.

### Stress

**Figure 6.**
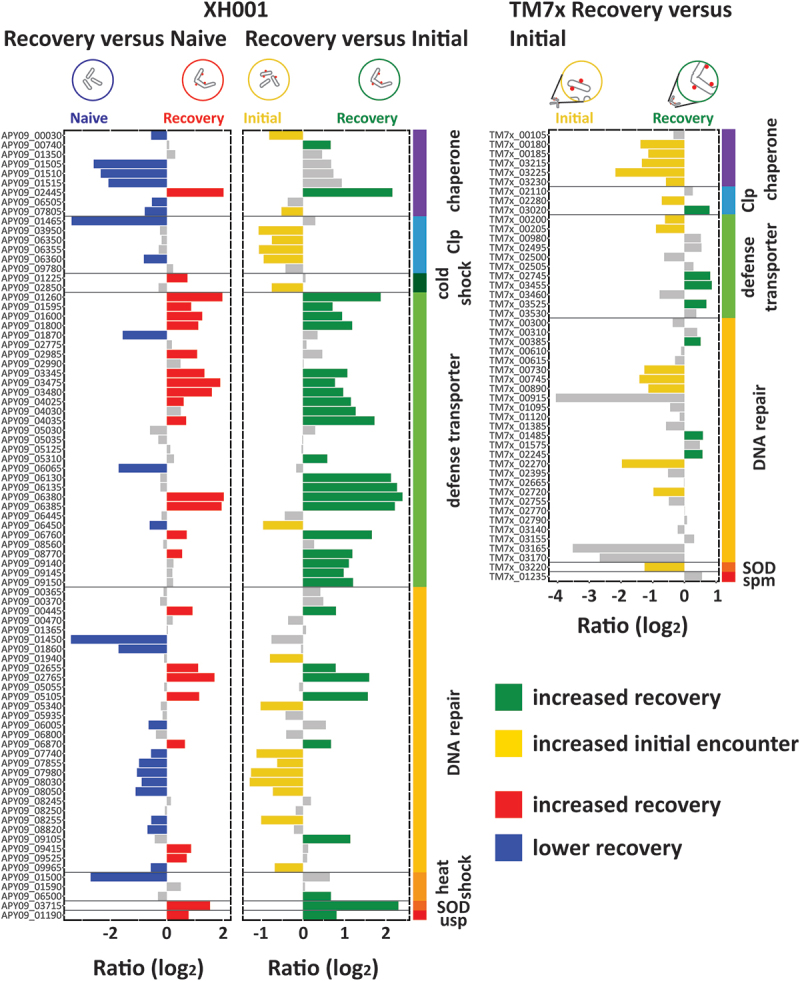
Stress. Genes involved in stress responses broken down into sub-categories chaperones, heat shock proteins, cold shock proteins, clp proteases, defense transporters, DNA repair, sodium oxide dismutase (SOD), universal stress protein (usp), and spermidine synthase (spm). Bar plots show the log_2_ ratio between the stable symbiosis coculture and the initial encounter coculture. Blue: decreased in XH001/TM7× versus XH001n; Green: increased in recovery; yellow: increased in initial encounter; Grey: statistically unchanged.

Previous studies indicated that TM7× epibiont association can be stressful for the host XH001 [11,12]. However, a detailed look at stress-associated genes by group function showed more specific changes (Figure 6). When comparing XH001 to XH001n the increased expression during recovery was primarily seen in the defense transporter genes with 14 out of 31 genes increased but only 3 decreased. Other genes with increased expression included a putative molecular chaperone (APY09_02445), a cold shock protein (APY09_01225), transcription-repair coupling factor (APY09_00445), DNA replication and repair gene *recF* (APY09_02655), methylated-DNA – protein-cysteine methyltransferase (APY09_05105), single-stranded DNA-binding proteins (APY09_06870, APY09_09415, APY09_09525) as well as sodium oxide dismutase (APY09_03715) and the universal stress protein (APY09_01190). Chaperones were primarily down compared to XH001n (6 out of 9 genes) while DNA repair trended downward (11 out of 30 genes decreased, 7 increased). Decreased genes included SsrA binding protein (APY09_00030), chaperones *grpE* (APY09_01510), *dnaK* (APY09_01515), *dnaJ* (APY09_06505), heat shock chaperone (APY09_07805), *clpB* (APY09_01465), *clpX* (APY09_06360), *recG* (APY09_08820), *recO* (APY09_06005), exonuclease ABC subunits B and C (APY09_08030, APY09_07980), DNA polymerase I (APY09_08050), DNA ligase A (APY09_09965), and a heat shock protein (APY09_01500). In the previous study, we also saw chaperones with lower expression compared to XH001n [11]. This might indicate lower levels of protein production in the presence of TM7×.

Comparing recovery phase XH001/TM7× to the initial encounter gave generally similar results to the XH001n comparison except for chaperones and clp proteases (Figure 6). The chaperones showed generally higher levels during recovery, though only *groES* (APY09_00740) and the putative molecular chaperone (APY09_02445) made the statistical cutoff. The clp proteases showed generally higher levels during the initial encounter, including *clpC* (APY09_03950), both putative *clpP* (APY09_06350, APY09_06355), and *clpX* (APY09_06360). Overall, 31 stress related genes were higher during recovery and 17 were higher during the initial encounter indicating somewhat higher levels of stress during the recovery phase.

For the TM7× epibiont genes, the results were generally consistent with reduced stress for TM7× (Figure 6). 14 stress related genes were higher during the initial encounter while only 7 were higher during the recovery phase. Genes expressed more during the initial encounter included most of the chaperones, several components for DNA repair, and sodium oxide dismutase (TM7×_03220). Genes higher during recovery were a predicted clp protease (TM7×_03020), 3 defense transporters, and 3 DNA repair protein genes including *recG* (TM7×_02245).

## Conclusion

The repeatability of the phases during TM7× interaction with naive XH001 was only recently established and this has provided a foundation to explore the transcriptional profile during these phases. The recovery phase marks a vital change in the epibiont and host interaction, from host killing to the growth of both species. Both bacteria appear to come to terms with living together, though what exactly determines that is not clear. We are early in the stages of characterizing this interaction which has significant effects on host numbers and physiology and thus implications for modulating levels of these bacteria *in vivo* and impacting the oral microbiome composition.

The study of the recovery phase is an accompaniment to our previous work with the initial encounter and stable symbiosis phases [11]. While the initial encounter showed limited expression changes, presumably due to the limited time for epibiont and host interaction, we saw extensive remodeling of the transcriptomes in both XH001 and TM7× during recovery and stable symbiosis. Some results were consistent across the later phases implying an importance in symbiosis rather than a phase specific role. TM7× showed increased levels for the arginine pathway and the arginine/ornithine antiporter, though XH001 did not give any indication of increased arginine production. TM7× also showed increased expression of pili genes which has recently been associated with host cell attachment for an environmental Saccharibacteria isolate [4]. Consistent with parasitism, XH001 showed increased stress gene expression during recovery and stable symbiosis phases. TM7× showed reduced expression, indicating that the association is beneficial for TM7× but stressful for the host.

Despite some consistencies, the transcriptome changes generally indicated that the episymbiotic association is a dynamic process demanding different responses at each phase. While transporters had extensive changes across the phases, highly expressed transporters varied greatly by phase, consistent with shifts in needs and substrate availability. Interestingly, XH001 showed lower expression of genes encoding cell cycle function during recovery despite the recovering cell numbers but slightly higher expression once stable symbiosis was established. However, DNA replication genes appeared down during stable symbiosis and we hypothesized that cell replication was lower and that the cell cycle expression might be responsible for the extensive elongated and clubbed end morphology adopted by XH001 [3]. TM7×’s type IV secretion system had higher expression during the initial encounter than during recovery indicating that it played a greater role early in the interaction. However, while there was no consistent change in the type IV secretory genes during stable symbiosis, three of the effector genes were higher in stable symbiosis than the initial encounter implying that these effectors may be important for long term association. Overall, the results thus far have provided valuable insights into the complex dynamics of episymbiosis and highlight the need for further research to fully understand the mechanisms behind this unique mode of bacterial interaction.

### Key messages:

The recovery phase marks an important shift in the TM7×/host interaction, switching from widespread killing of the host XH001 cells to an interaction where the host can survive and grow in the presence of epibiont, necessary for stable symbiosis.

Epibiont TM7× and its host XH001 extensively alter their transcriptomes during the recovery phase of epibiont/host association.

The recovery phase is marked by changes maintained in stable symbiosis as well as changes specific to the recovery phase.

## Supplementary Material

supplemental_Table_2.xlsx

supplemental_Table_1.xlsx

## Data Availability

The data discussed in this publication have been deposited in NCBI’s Gene Expression Omnibus [22] and are accessible through GEO Series accession number GSE196744 (https://www.ncbi.nlm.nih.gov/geo/query/acc.cgi?acc=GSE196744). Supplemental tables at (https://github.com/mcleanlab/TM7x_Host_Association_Study).
